# Corneal endothelial cell density and morphology in normal Iranian eyes

**DOI:** 10.1186/1471-2415-6-9

**Published:** 2006-03-06

**Authors:** Mohammad Nasser Hashemian, Sasan Moghimi, Masood Aghsaie Fard, Mohammad Reza Fallah, Mohammad Reza Mansouri

**Affiliations:** 1 Associated Professor of Ophthalmology, Tehran University Eye Research Center, Farabi Eye Hospital, Quazvin Sq., Tehran, Iran; 2 Assistant Professor of Ophthalmology, Tehran University Eye Research Center, Farabi Eye Hospital, Quazvin Sq., Tehran, Iran; 3 Resident of Ophthalmology, Tehran University Eye Research Center, Farabi Eye Hospital, Quazvin Sq., Tehran, Iran; 4 Assistant Professor of Ophthalmology, Tehran University Eye Research Center, Farabi Eye Hospital, Quazvin Sq., Tehran, Iran; 5 Associated Professor of Ophthalmology, Tehran University Eye Research Center, Farabi Eye Hospital, Quazvin Sq., Tehran, Iran

## Abstract

**Background:**

We describe corneal endothelial cell density and morphology in normal Iranian eyes and compare endothelial cell characteristics in the Iranian population with data available in the literature for American and Indian populations.

**Methods:**

Specular microscopy was performed in 525 eyes of normal Iranian people aged 20 to 85 years old. The studied parameters including mean endothelial cell density (MCD), mean cell area (MCA) and coefficient of variation (CV) in cell area were analyzed in all of the 525 eyes.

**Results:**

MCD was 1961 ± 457 cell/mm^2 ^and MCA was 537.0 ± 137.4 μm^2^. There was no statistically significant difference in MCD, MCA and CV between genders (*Student t-test*, P = 0.85, *P *= 0.97 and *P *= 0.15 respectively). There was a statistically significant decrease in MCD with age (*P *< 0.001, *r *= -0.64). The rate of cell loss was 0.6% per year. There was also a statistically significant increase in MCA (*P *< 0.001,*r *= 0.56) and CV (*P *< 0.001, *r *= 0.30) from 20 to 85 years of age.

**Conclusion:**

The first normative data for the endothelium of Iranian eyes seems to confirm that there are no differences in MCD, MCA and CV between genders. Nevertheless, the values obtained in Iranian eyes seem to be different to those reported by the literature in Indian and American populations.

## Background

Monolayer of corneal endothelial cell covers the posterior surface of descemet's membrane. In normal cornea dimension of endothelial cells are uniform. Corneal endothelium is metabolically active and responsible for keeping the corneal stroma in its usual dehydrated state of 70% water [1-3].

Prior to 1970s, the study of corneal endothelium was limited to biomicroscopic evaluation for guttata, fold and keratic precipitates. Nowadays specular microscope has made the evaluation of endothelium possible. The aim of quantitative specular microscopic analysis is to assign values to endothelial cells that can provide a measure of their functional states. It makes the measurement of mean cell density (MCD), mean cell area (MCA) possible as well as measurement of variations in cell size (polymegathism) and cell shape (polymorphism). The specular microscope has been used to establish and compare normative data for endothelium parameters among ethnic groups [4-6] as well as sexes [4,5,7]. These parameters provide an index of the functional capacity of the endothelium. Normative data regarding endothelial cell density and morphology are thus important because they facilitate assessment of the functional reserve of the endothelium in individual patients.

Although several studies have also shown a clear inverse relationship between MCD and age in normal populations, [7-9] other investigators have shown that there is no significant correlation between MCD and age in populations aged 40 and above [5]. Moreover some data have shown statistically significant differences in endothelial parameters between sexes [4,5,7].

Because of difference in endothelial parameters among various populations, study of normative data of each population is important. A comparison of endothelial cell densities in the American, Japanese and Indian populations, [6,7] revealed a significantly higher value in the Japanese population and lower in the Indians. The corneal endothelial cell density and morphology in the Iranian population, which may differ from those in other races, have not been documented so far. This study describes the endothelial cell density and morphology in normal Iranian eyes in relation to age and gender and reports the rate of endothelial cell loss with increasing age.

## Methods

A total of 525 eyes from 525 normal volunteers (right eye for each one) aged 20 to 85 years of age were examined. The subjects of the study were randomly selected among the center's staff, visitors, and outpatients of Farabi Eye Hospital. All volunteers signed an informed consent.

Sample size for MCD comparison between male and female subjects was calculated using a minimum detectable difference of 100 cells/mm^2^, α error of 0.05, and a power of 0.80. Exclusion criteria included history of intraocular surgery or ocular trauma, increased intraocular pressure, uveitis, corneal opacity, evidence of endothelial dystrophy on slit-lamp biomicroscopy and diabetes mellitus.

After routine ophthalmic examination, all volunteers underwent specular microscopy using a noncontact specular microscopy (SP2000: Topcon corporation, Japan). A single examiner performed all measurements. The procedure for specular microscopy was as follow: three images from central cornea were taken of at least 50 contiguous cells and were manually marked with a mouse by the examiner for analysis by a built-in software program.

The computer automatically evaluated, calculated and displayed mean cell density (cell/mm^2^) mean cell area (μm^2^), and coefficient of variation (CV) in cell size. The CV in cell size (standard deviation divided by the mean cell area) was used as an index of the extent of variation in the cell area (polymegathism). The mean of each variable from three image of central cornea was used as MCD, MCA and CV.

Data were analyzed using the SPSS statistical program (version 10). Mean arithmetic differences, standard deviations, and correlation coefficients between observations were calculated. Regression analysis was used to determine the changes in endothelial cell characteristics with age controlled by sex. Student t-test was used to compare endothelial cell parameters with sex and race.

## Results

The patient population consisted of 525 volunteers aged 20 to 85 years old (mean age 52.7 ± 19.1 years) of whom 271(51.7%) were male and 254 (48.3%) were female. Mean best-corrected visual acuity was 20/24 (range, 20/200 to 20/20) with a mean spherical equivalent of +0.63 (ranged, -8.5 to +4.5)

The MCD of the population was 1961 ± 457 cell/mm^2 ^(range, 1030 to 3341). The MCA was 537.0 ± 137.4 μm^2 ^(range, 170 - 976μm^2^). The mean CV was 24.1 ± 7.1 (range, 2.8 to 57). There were no statistically significant differences in MCD, MCA and CV between genders. (*Student t-test*, P = 0.85, *P *= 0.97 and *P *= 0.15 respectively).(Table 1)

**Table 1:** Endothelial cell characteristics of the study population in different sexes

	**Male**	**Female**	***P*value**†	**Total**
No. of eyes	271 (51.7%)	254 (48.3%)		525
Age (year) *	51.9 ± 19.6	53.6 ± 18.6	0.32	52.7 ± 19.1
Mean cell density* (cells per mm^2^)	1,965 ± 471	1,958 ± 443	0.85	1,961 ± 457
Mean cell area (μm^2^) *	537.1 ± 140.8	536.8 ± 133.9	0.97	537.0 ± 137.4
Coefficient of variation in cell size (%)*	54.6 ± 7.4	23.7 ± 6.7	0.15	24.1 ± 7.1

* Mean ± SD

† Student t-test

Results of MCD, MCA and CV for different age groups are listed in table 2. There was a statistically significant decrease in MCD with increase in age (*P *< 0.001, *r *= -0.64). Regression analysis indicated a cell loss rate of 0.6% per year (12.8 ± 0.69 per year). A significant increase in mean cell area (*P *< 0.001,*r *= 0.56), and CV in cell size (*P *< 0.001, *r *= 0.30) with age was noted.

**Table 2:** Endothelial cell characteristics of the study population in different age groups

Age group	No. of eyes	Mean cell density (cells / mm^2^) *	Mean cell area (μm^2^) *	Coefficient of variation in cell size (%)*
20–30	102	2,407 ± 399	427.8 ± 74.9	20.4 ± 5.5
31–40	45	2,245 ± 349	458.9 ± 81.0	23.1 ± 7.2
41–50	66	2,071 ± 340	496.5 ± 102.6	24.2 ± 7.8
51–60	87	1,939 ± 344	535.7 ± 106.6	24.1 ± 6.6
61–70	122	1,775 ± 348	584.7 ± 127.9	25.5 ± 7.5
>70	103	1,571 ± 328	649.6 ± 147.3	26.8 ± 6.4

* Mean ± SD

In this series, there was a consistent decrease in MCD (*r *= -0.50) and a corresponding increase in MCA (*r *= 0.45) between 20 and 60 years of age followed by a substantial decrease in correlation for MCD (*r *= -0.30) and MCA (*r *= 0.25) in the older age groups. The endothelial cell counts in the study population were also compared with previously described values for the Indian and American populations [6]. (Table 3)

**Table 3:** Comparison of endothelial cell density in Iranian, American, and Indian eyes

**Age group (year)**	**Iranian population**		**American population**		**Indian population**	
	
	No. of eyes	Cell density (cells/mm^2^) *	No. of eyes	Cell density (cells/mm^2^) *	No. of eyes	Cell density (cells/mm^2^) *
20–30	102	2,407 ± 399	11	2,977 ± 324†	104	2,782 ± 250†
31–40	45	2,245 ± 349	6	2,739 ± 208†	96	2,634 ± 288†
41–50	66	2,071 ± 340	11	2,619 ± 321†	97	2,408 ± 274†
51–60	87	1,939 ± 344	13	2,625 ± 172†	98	2,438 ± 309†
61–70	122	1,775 ± 348	8	2,684 ± 384†	88	2,431 ± 357†
>70	103	1,571 ± 328	15	2,431 ± 339†	54	2,360 ± 357†

* Mean ± SD

† Statistically significant by Student t-test

## Discussion

Endothelial cell analysis provides important clinical information on corneal function and viability. The determination of the endothelial cell density (ECD) has become an accepted practice both clinically and in research to provide information on the cell layer needed to maintain corneal transparency [10]. The potential clinical uses include the assessment of the endothelium in donor corneas, the monitoring of different anterior segment surgery techniques, and the longitudinal effects of intraocular surgery, such as cataract surgery or implantation of phakic intraocular lenses [10-13]. When performing intraocular procedures, endothelial trauma should be minimized, and specular endothelial microscopy is recognized as being essential in evaluating the safety of new intraocular or corneal surgical procedures and intraocular lenses [10-13].

Many studies have been published on the relationship of endothelial cell density and morphology with age, gender, and ethnicity. Although investigators differ in their findings about the relationship of age and gender to endothelial characteristics, it is clear that significant differences in corneal endothelial properties do exist among races and ethnic groups [4-7].

Decisions concerning endothelial health and function in an individual should be based on normative data derived from the underlying population. This study provides data on endothelial cell characteristics in the normal Iranian population.

Noncontact endothelial imaging reduces the risk of corneal epithelial damage, artifacts due to corneal manipulation, and transmission of infection. The disadvantage of this method is less control over patient eye movement, and therefore less resolution and magnification [14]. The recommended sample size for endothelial analysis is 75–100 cells [3,15]. However, a sample size of 50 cells may be adequate to study a normal cornea that does not have excessive pleomorphism [16,17]. Many studies have compared contact and non-contact specular microscopy and have shown that contact specular microscopy is accurate and reproducible in the determination of endothelial cell density [10,14,17]. The average number of cells analyzed per eye in this study was 68.5 ± 6.8.

The mean endothelial cell density in this study was within the range described for normal corneas [18]. In our study, the cell loss rate with age (0.6% per year) was more than that described in most previous studies (0.3–0.5% per year) [19,20], even as in longitudinal studies in which some subjects are examined again at a later date, a higher annual loss rate was reported (0.3–1.1% per year) [21].

The results of this investigation have shown that with increasing age there is a general trend toward decreased MCD, increased MCA, and increased CV in cell size. These findings are in agreement with many previous investigations [7-9,19].

Although several studies have reported a clear inverse relationship between MCD and age in normal populations, [7-9] other investigators have stated that there is no significant correlation between MCD and age in populations aged 40 and above. Padilla et al showed that although there was a definite trend toward decreasing MCD and MCA with increasing age up to sixth decade of life, but a reverse trend was noted at 61 and above [4]. Significant increase of polymegathism from the fourth decade has been attributed to these findings [4,5,7].

There are conflicting reports about the relationship between gender and endothelial cell characteristics. Some studies showed differences between sexes, [4,5] but others did not find any statistically significant differences between them [6,8,14,22]. Our data showed no statistically significant differences in MCD, MCA and CV between sexes.

The cell density in Iranian eyes in this study was significantly less than that reported in American eyes by Matsuda et al [6] and Indian eyes by Rao et al. [7] The differences were statistically significant in all age groups [6,7].

Some studies have reported a high MCD (>3800 cells/mm^2^) in children, but revealed an unexpected extent of non-uniformity in cell shape (pleomorphism) in the endothelia of normal healthy children [23]. In this study, volunteers aged less than 20 years were not included and therefore their characteristics could not be reported.

The lower incidence of pseudophakic bullous keratopathy in Japanese eyes may be due to their higher endothelial cell density [6]. Accordingly, the reduced endothelial counts in Iranian eyes may well predispose this population to an increased risk of pseudophakic bullous keratopathy.

## Conclusion

In conclusion, this is the first report of endothelial cell characteristics in the normal Iranian population.

Our study showed no statistically significant difference in endothelial cell density between genders. A consistent decrease in MCD and increase in MCA with age was shown up to age 60, after which the correlation between age and these parameters decreases. Comparison with previous studies indicates that endothelial cell density in the Iranian population is less than that in the Indian and American populations. Our study may confirm the lowest density reported to date.

### Ethic approval

The review board and ethical committee of Eye Research Center of Tehran University of Medical Sciences approved the trial.

### Informed consent

Written Informed consent was obtained from all the volunteers after complete explanation.

## Competing interests

The author(s) declare that they have no competing interests.

## Pre-publication history

The pre-publication history for this paper can be accessed here:



## References

[B1] Stocker FW (1953). The endothelium of the cornea and its clinical implications. Trans Am Ophthalmol Soc.

[B2] Mishima S (1982). Clinical investigations on the corneal endothelium. Ophthalmology.

[B3] Waring GO, Bourne WM, Edelhauser HF, Kenyon KR (1982). The corneal endothelium: normal and pathologic structure and function. Ophthalmology.

[B4] Padilla MD, Sibayan SA, Gonzales CS (2004). Corneal endothelial cell density and morphology in normal Filipino eyes. Cornea.

[B5] Snellingen T, Rao GN, Shrestha JK, Huq F, Cheng H (2001). Quantitative and morphological characteristics of the human corneal endothelium in relation to age, gender, and ethnicity in cataract populations of South Asia. Cornea.

[B6] Matsuda M, Yee RW, Edelhauser HF (1985). Comparison of the corneal endothelium in an American and a Japanese population. Arch Ophthalmol.

[B7] Rao SK, Ranjan Sen P, Fogla R, Gangadharan S, Padmanabhan P, Badrinath SS (2000). Corneal endothelial cell density and morphology in normal Indian eyes. Cornea.

[B8] Laing RA, Sandstrom MM, Berropsi AR (1976). Changes in corneal endothelium as a function of age. Exp Eye Res.

[B9] Laule A, Cable MK, Hoffman CE, Hanna C (1978). Endothelial cell population changes of human cornea during life. Arch Ophtalmol.

[B10] American Academy of Ophthalmology (1997). Corneal endothelial photography. Three-year revision. Ophthalmology.

[B11] Bourne WM, Nelson LR, Hodge DO (1994). Continued endothelial cell loss ten years after lens implantation. Ophthalmology.

[B12] Walkow T, Anders N, Klebe S (2000). Endothelial cell loss after phacoemulsification: relation to preoperative and intraoperative parameters. J Cataract Refract Surg.

[B13] Pop M, Payette Y (2004). Initial results of endothelial cell counts after Artisan lens for phakic eyes. Ophthalmology.

[B14] Landesz M, Siertsema JV, Van Rij G (1995). Comparative study of three semiautomated specular microscopes. J Cataract Refract Surg.

[B15] American Academy of Ophthalmology (1991). Ophthalmic Procedures Assessment. Corneal endothelial photography. Ophthalmology.

[B16] Olsen T (1979). Optical principles for estimation of endothelial cell density with the noncontact specular microscope. Acta Ophthalmol.

[B17] Hirst LW, Ferris FL, Stark WJ, Fleishman JA (1980). Clinical specular microscopy. Invest Ophthalmol Vis Sci.

[B18] Hirst LW, Ferris FL, Stark WJ, Fleishman JA (1980). Normal endothelial cell count range. Ophthalmology.

[B19] Yee RW, Matsuda M, Schultz RO, Edelhauser HF (1985). Changes in the normal corneal endothelial cellular pattern as a function of age. Curr Eye Res.

[B20] Carlson H, Bourne WM, McLaren JW, Brubaker RF (1988). Variations in human endothelial morphology and permeability to fluorescein with age. Exp Eye Res.

[B21] Cheng H, Jacobs PM, McPherson K, Noble MJ (1985). Precision of cell density estimates and endothelial cell loss with age. Arch ophrhalmol.

[B22] Inoue K, Tokuda Y, Inoue Y, Amano S, Oshika T, Inoue J (2002). Corneal endothelial cell morphology in patients undergoing cataract surgery. Cornea.

[B23] Müller A, Doughty MJ, Wright L (2000). Reassessment of the corneal endothelial cell organisation in children. Br J Ophthalmol.

